# Burnout and Physical Symptoms in Healthcare Professionals: A Cross‐Sectional Study

**DOI:** 10.1002/nop2.70379

**Published:** 2025-11-23

**Authors:** Núria Puigtió‐Rebollo, Bernat Carles Serdà‐Ferrer, Miquel Sitjar Suñer, Mariano Gacto‐Sánchez

**Affiliations:** ^1^ University School of Health and Sport (EUSES), University of Girona Girona Spain; ^2^ Nursing Department University of Girona Girona Spain; ^3^ Faculty of Medicine CEIR Campus Mare Nostrum (CMN), Instituto Murciano de Investigación Biosanitaria‐Virgen de la Arrixaca (IMIB‐Arrixaca), University of Murcia Murcia Spain

**Keywords:** Burnout syndrome, healthcare professional, nursing, physical symptoms

## Abstract

**Aim:**

The Burnout syndrome (BOS) generates sustained stress and an inability to cope with demands. The aims of this cross‐sectional study were (1) to describe the prevalence of BOS; (2) to explore the relation of the BOS with physical symptoms; and (3) to determine the profile favouring the development of the BOS.

**Design:**

A cross‐sectional descriptive study.

**Methods:**

The study was conducted among a sample of 759 healthcare professionals. Data on sociodemographic variables, physical symptoms, the Maslach Burnout Inventory, Cervical Disability Index and Numeric Pain Rating Scale were collected.

**Results:**

The prevalence of BOS was high, since more than one out of two participants experienced the syndrome. Mild–moderate levels of burnout were the most prevalent, whilst a low percentage of healthcare professionals displayed signs of severe burnout. High Emotional Exhaustion was the most common dimension, followed by high Depersonalisation, and low levels of Personal Fulfilment. Roughly three out of four participants experienced muscle pain. A nurse of young age working in a hospital or an emergency department emerges as a specific vulnerable profile.

**Conclusion:**

The high prevalence of BOS detected among healthcare professionals propels stakeholders to take proactive actions to prevent and revert this health condition, especially considering the profile of nurses at a young age working at a hospital or emergency department, since this specific profile is at a higher risk. The current study confirms the need to prevent and overcome BOS by means of developing and implementing multidimensional tailored interventions to decrease and/or revert symptoms, including psychological aspects associated with the syndrome itself, alongside decreasing unspecific muscle pain in the vertebral region.

**Patient or Public Contribution:**

No patient or public contribution.

**Implications for the Profession and/or Patient Care:**

Gender is not a specific predictor of the development of BOS. Nurses usually show high Emotional Exhaustion and low Personal Fulfilment. The presence of BOS is also associated with muscle pain. Advocacy on prevention, assessment and screening of BOS in the field of healthcare is of paramount importance.

**Impact:**

Proactive actions shall be adopted to prevent and overcome BOS in healthcare professionals by means of developing and implementing multidimensional tailored interventions to decrease and/or revert symptoms including psychological aspects associated to the syndrome alongside decreasing unspecific muscle pain in the vertebral region.

## 1 Introduction

The Burnout syndrome (BOS) can be defined as a psycho‐emotional disorder generating sustained stress over time, and inability to cope with the demands from the professional environment in an adaptive manner (Schaufeli and Greenglass 2001; Zarei et al. 2019). In late stages, burnout may even lead to physical problems, including pain, since the induced stress causes the muscles to be in a state of guardedness: this may trigger other reactions of the body and even promote stress‐related disorders (Chen‐Lim et al. 2022; De Hert 2020). Typical places the body holds tension include the shoulders, neck, head and back (Chen‐Lim et al. 2022; De Hert 2020). The incidence and prevalence of the BOS is highly variable (Baro Vila et al. 2022; Liu et al. 2020; Wang et al. 2020). In USA, the prevalence of the BOS among professionals in Primary Care corresponded to 52.9% (Zarei et al. 2019) whilst it was of 60.3% among Resident Trainees (Dyrbye et al. 2014). Around 46.5% of the professionals involved in the areas of Intensive Care also suffered from BOS before the outbreak of the COVID‐19 pandemic period (Embriaco et al. 2012). Furthermore, nurses' burnout scores did not differ significantly before and during the COVID‐19, fact that highlights the ongoing challenges faced by nurses in their clinical routines (Rizzo et al. 2023).

## 2 Background

The risk of experiencing BOS is higher in those healthcare professionals focusing their professional activity in the approach and treatment of patients in a direct manner (Liu et al. 2020). Gil Monte and Peiró defined the specific professional stress factors affecting HCPs. Those were: shift‐working, being ‘on call’ for emergencies, lack of professional staff, treating patients at high‐risk, direct contact with specific conditions, pain, death, lack of autonomy to decide and technological advances with short timeframes for adaptation. The pressure on HCPs during the COVID‐19 pandemic generated an increase of the professional stress factors on the different strata of HCPs (Soto‐Rubio et al. 2020).

Research developed during the Ebola pandemic led to the ascertainment of a relationship between stress and gender among HCPs, since higher figures of incidence of stress were stated among women (Shultz et al. 2016). According to the professional field of activity, nurses were the healthcare stratum with the highest impact, due to their close contact with patients (Chen‐Lim et al. 2022; Soto‐Rubio et al. 2020; Zhang et al. 2021).

Previous literature identified high figures of BOS among HCPs in Intensive Care and Emergency Units, therefore confirming the need to urgently focus on tailored interventions to decrease professional exhaustion (Gualano et al. 2021; Surendran et al. 2024). Besides its impact on personal or professional spheres, BOS may increase the risk of developing physical symptoms (pain in the upper limb, cervical and/or lumbar vertebral regions) (Balagué et al. 2012). Furthermore, unspecific muscle pain in the vertebral region shows a strong relation with stress and anxiety (Balagué et al. 2012; Ortego et al. 2016). From a Physical Therapy perspective, the physical symptoms stemming from the BOS may be therapeutically approached but, moreover, several psychological symptoms as stress, anxiety and depression may also be identified and addressed (Bamforth et al. 2023; Ortego et al. 2016); physical symptoms, as well as muscular pain in the professional environment may also be identified as factors causing BOS (Roslan et al. 2021). The psychosomatic research performed in the field of BOS has already stated the underlying correlation between BOS and psychological and psychosomatic symptoms among nurses (Ali and Eissa 2018). More recently, shortened scales taking psychosomatic symptoms into account have been developed and proven effective in resource management as well as in the assessment of BOS among nurses (Ivánkovits et al. 2024).

Gender, age, job category and the professional setting may have an impact on the degree of BOS that the staff may experience (Wu et al. 2021; Zhou et al. 2022). Within this framework, the key‐role of the gender in the high association with high levels of the three domains of burnout has been highlighted (Chen et al. 2021; Roslan et al. 2021). Moreover, being younger is also a significant factor related to the development of BOS in HCPs (López‐López et al. 2019; Roslan et al. 2021). Mental health disorders in HCPs may have a fundamental impact on the quality of life and may subsequently entail a negative impact on the quality of healthcare itself, as well as on the competences on clinical reasoning, therefore hindering the adequate control of the pandemic situation (Giordano et al. 2022).

This study aims to address a critical gap in knowledge by exploring the co‐occurrence of BOS with physical symptoms (such as muscle pain) among HCPs. Additionally, the study seeks to identify specific healthcare profiles and work environment factors that may contribute to increase the risk of developing BOS. By examining these elements, this framework could provide valuable insights into the improvement of both the physical and mental health dimensions of healthcare workers, ultimately enhancing the overall well‐being within the healthcare system.

## 3 The Study

### 3.1 Aims and Objectives

The main aim of the current research is to describe the prevalence of BOS in Spain, alongside exploring the relation of BOS with physical symptoms among a sample of HCPs. A second aim is to determine the specific profiles or characteristics favouring the development of BOS among HCPs.

## 4 Methods

### 4.1 Study Design, Setting and Participants

A cross‐sectional descriptive study was conducted among a sample of HCPs and students undergoing practical internships from the Degrees in Medicine, Nursing and Physical Therapy in Spain. The sample was recruited by means of non‐probabilistic convenience sampling. Inclusion criteria were being a healthcare professional and/or student within a practical internship period in a health‐related Degree. The exclusion criteria were being on sick leave, not understanding Spanish, working abroad of Spain and being under 18 years old.

### 4.2 Data Collection

The sample was recruited from 7 May to 15 June 2021 throughout three different means: (1) contacting the responsible person from different healthcare institutions from Catalonia and other regions of Spain for him/her to disseminate the recruitment information within the corresponding healthcare institution; (2) contacting through Linkedin; (3) the departments of Medicine, Nursing and Physiotherapy from the University of Girona (Girona, Spain) were contacted for possible voluntary participation from students undergoing practical internship periods.

### 4.3 Ethical Considerations

The study was carried out following the principles of the Declaration of Helsinki. The experimental protocols were approved by the international review board at the Dr. Josep Trueta University Hospital of Girona (approval‐number 2021.052). All participants received information on the study previously, alongside the corresponding Information Sheet and Informed Consent, and the possibility of withdrawing from the study was also highlighted across the study process. The informed consent was obtained from all participants.

### 4.4 Measures

Different variables were collected throughout a tailored on‐line questionnaire created ad‐hoc. The Informed Consent, Information Sheet and the ad‐hoc questionnaire were developed in Spanish, since the validated Spanish version of the scales Maslach Burnout Inventory (MBI) and Cervical Disability Index (CDI) were used. Data on three groups of variables were therefore collected.

#### 4.4.1 Sociodemographic Variables

The variables included within the sociodemographic group were age, gender, civil status, number of children, region of residence, healthcare field, type of institution in which the participant worked, seniority in his/her current institution, and seniority as a healthcare professional overall. Whenever the participant was a student undergoing a practical internship period, he/she was asked on the number of practical hours developed in the current field alongside the total number of practical hours developed overall during the whole period of studies. Age was therefore categorised in four different groups (< 30; 30–39; 40–49; > 50), as stated elsewhere (Cunill et al. 2020). BOS‐related variables and variables on physical symptoms (pain intensity and degree of cervical disability) were also collected.

#### 4.4.2 BOS‐Related Variables

Maslach Burnout Inventory‐Human Services Survey (MBI‐HSS): The Spanish version (Gil‐Monte 2005) of the original questionnaire (Maslach and Jackson 1986) was used. This questionnaire presents three different dimensions:

- Emotional Exhaustion (EE): Participant's feelings on being emotionally overwhelmed by his/her work‐related situation. This dimension includes nine items; the score is direct to the intensity of the syndrome. The total score range is 0 to 54 points. The cut‐off points corresponding to this dimension are 18 and 26, so that ‘low’ corresponds to scores below 18 points, ‘mild’ to the score range 19–26 and ‘high’ from 27 points on.
- Depersonalisation (DP): Cold and impersonal response towards patients. This dimension includes five items. The score is also directly proportional to the intensity of the syndrome. The total score encompasses a 0–30 range. The corresponding intervals are low (5 points or below), mild (from 6 to 9 points) and high (10 points and above).
- Personal Fulfilment (PF): Feelings of incompetence and inefficiency at work. It consists of eight items. The score is inversely proportional, so that low scores entail higher figures of BOS. The scores range from 0 to 48, with the following levels: low (below 33 points), mild (from 34 to 39 points) and high (above 40 points).

The aforementioned dimensions are independent, since the scores are not combined into a sole total score. Thus, the BOS may be defined through high scores in the EE and DP dimensions, and low scores in the PF dimension. BOS is therefore ‘present’ whenever one of the three dimensions is positive, i.e., whenever one of the three dimensions is either high (EF, DP) or low (PF). In the current study, due to the high scores of positive dimensions across the different participants, and based on the study by Liu et al., BOS has been categorised into four different levels: no BOS (three dimensions are negative), mild BOS (solely one positive dimension), moderate BOS (two positive dimensions) and severe BOS (the three dimensions are positive) (Liu et al. 2020). The psychometric properties on internal consistency are moderate‐to‐good for the three dimensions, since Cronbach's alpha for PF, EE and DP corresponds to 0.71, 0.85 and 0.58, respectively (Gil‐Monte 2005).

#### 4.4.3 Physical Symptoms

##### 4.4.3.1 Register for Physical Symptoms

The participant highlights the presence of any of the following physical symptoms: unspecific muscular pain, cephalea, vertigo and an open question on the presence of any other physical symptom. In order to identify possible relationships between psychological and physical symptoms, the results will be drafted and compared to those from the BOS (Cunill et al. 2020).

##### 4.4.3.2 Cervical Disability Index (CDI)

Questionnaire validated in Spanish measuring the impact that cervical pain may have on activities of daily living (ADLs) (Vernon 2008). The questionnaire includes 10 questions corresponding to ADLs: pain intensity, personal hygiene, lifting objects, reading, cephalea, concentration, working, driving, sleeping and leisure activities. Each question has six response options corresponding to six different levels of cervical disability. The scores range from 0 (no disability) to 5 (maximal disability). The total score is expressed as a percentage: the different scores (in a 0–50 range) are added and then multiplied by two into a total score in a 0–100 range. The higher the CDI score, the higher the level of disability. In this study, the variable was subsequently categorised into four levels: 0–4 corresponds to lack of disability, whereas a score between 5 and 14 represents mild disability, 15–24 points entail moderate disability, a 25–34 range corresponds to severe disability and scoring above 35 points is linked to complete disability. This categorisation into four different levels of the CDI has been methodologically used in different previous studies (MacDermid et al. 2009; Vernon 2008). Unidimensional disability instrument for patients with neck pain. The CDI is a unidimensional instrument, with values of Cronbach's alpha generally exceeding the threshold of 0.85, which entails excellent internal consistency (MacDermid et al. 2009).

##### 4.4.3.3 Numeric Pain Rating Scale (NPRS)

It is used to measure the perception and intensity of pain in the vertebral spine, across the cervical, thoracic and lumbar regions. The following categories were created: no pain (0), mild pain (1–3), moderate pain (4–6) and severe pain (7–10) (Jensen and McFarland 1993). Cronbach's alpha for the NPRS is excellent (0.89) in a general population of healthy adults (Herr et al. 2004).

### 4.5 Statistical Analyses

A descriptive analysis was initially performed: percentages and absolute frequencies were used to describe the sociodemographic characteristics of participants, alongside the BOS‐related variables and physical symptoms. To assess the degree of cervical disability and the intensity of pain, means and standard deviations were used. Chi‐squared tests were used to evaluate the BOS by gender, by groups of HCPs, by physical symptoms, and by the presence of unspecific muscle pain in the cervical spine. Student t‐tests were applied to compare the mean of incidence of each one of the three BOS dimensions across HCPs. After testing for normality, differences across the means of each BOS dimension between genders were assessed through the Kruskal–Wallis test, whereas a one‐factor ANOVA was used to compare differences in the intensity of BOS on the degree of pain intensity and the extent of cervical disability. The Pearson correlation coefficient was applied to analyse possible linear relationships between the degree of cervical disability and the scores of the different BOS dimensions.

Even though the study is cross‐sectional, sociodemographic characteristics were figures previous to the administration of the questionnaire, so that, with the aim of determining possible associations involved in the development of different level of burnout, a multinomial logistic regression model was used (enter method), stating the absence of burnout as the reference category. The level of statistical significance was set at 0.05 (95% CI) throughout all the analyses performed. All data were analysed using SPSS version 28.0 (IBM, Armonk, NY, USA).

## 5 Results

### 5.1 Participant Characteristics

A total of 803 responses were initially collected. After applying eligibility criteria, a total of 44 potential participants were excluded: among them, six were not on active duty, whereas nine were professionally working abroad, and 29 corresponded to duplicated responses, detected through the Google application (from the ‘remove duplicates window’, selecting the columns included in the search for duplicate data).

The final sample was therefore composed of 759 participants (521 women; 68.6%). The mean age (standard deviation) was 36.7 (10.7) years. When clustering per age‐ranges, 240 (32.3%) participants were below 30 years of age, whilst 236 (31.8%) corresponded to a 31–39 age range, 160 (21.5%) were comprised between 40 and 49 years of age, and 116 (the extant 14.4%) were beyond 50 years of age. Based on the region of residence, 408 participants (53.8%) corresponded to the region of Catalonia. As for the health profession, 283 (37.3%) were nurses, 162 (21.3%) were physical therapists, whereas 107 (14.1%) were physicians, 57 (7.5%) were nursing assistants, 50 (6.6%) were students undergoing practical internships and the extant 100 participants (13.2%) corresponded to several and heterogeneous health professions. A total amount of 416 (54.80%) participants had over 10 years of professional experience. Further sociodemographic data are shown in Table 1.

**TABLE 1:** Sociodemographic characteristics of the sample (*N*, %).

Sociodemographic characteristics	Total (*N* = 759)	No BOS (*n* = 312)	Mild BOS (*n* = 242)	Moderate BOS (*n* = 133)	Severe BOS (*n* = 72)
Gender					
Women	521 (68.6)	196 (62.8)	195 (80.6)	79 (59.4)	51 (70.8)
Men	238 (31.4)	116 (37.2)	47 (19.4)	54 (40.6)	21 (29.2)
Age (*n* = 743)					
≤ 30 years	240 (32.3)	81 (26.0)	69 (28.5)	44 (33.1)	27 (37.5)
31–39 years	236 (31.8)	94 (30.1)	85 (35.1)	48 (36.1)	28 (38.9)
40–49 years	160 (21.5)	79 (25.3)	42 (17.4)	28 (21.1)	11 (15.3)
≥ 50 years	107 (14.4)	53 (17.0)	38 (15.7)	11 (8.3)	5 (6.9)
Civil status (*n* = 747)					
Single	298 (39.9)	111 (35.6)	92 (38.0)	61 (45.9)	34 (47.2)
Married	287 (38.4)	134 (42.9)	91 (37.6)	40 (30.1)	22 (30.6)
In a relationship	131 (17.6)	56 (17.9)	40 (16.5)	25 (18.8)	12 (16.7)
Divorced	25 (3.3)	8 (2.6)	12 (5.0)	3 (2.3)	2 (2.8)
Widowed	6 (0.8)	1 (0.3)	4 (1.7)	1 (0.8)	0 (0)
Region of residence					
Catalonia	408 (53.7)	177 (56.7)	123 (50.8)	69 (51.9)	39 (54.2)
Madrid	100 (13.2)	33 (10.6)	37 (15.3)	21 (15.8)	9 (12.5)
Rest of Spain	251 (33.1)	102 (32.6)	82 (32.8)	42 (32.3)	24 (33.3)
Professional activity					
Nursing	283 (37.3)	90 (28.8)	116 (47.9)	39 (29.3)	38 (52.8)
Physical Therapy	162 (21.3)	102 (32.7)	37 (15.3)	18 (13.5)	5 (6.9)
Medicine	107 (14.1)	39 (12.5)	30 (12.4)	29 (21.8)	9 (12.5)
Nursing Assistant	57 (7.5)	20 (6.4)	16 (6.6)	15 (11.3)	6 (8.3)
Emergency Medical Technician	44 (5.8)	22 (7.1)	6 (2.5)	11 (8.3)	5 (6.9)
Students under Practical Internship	50 (6.6)	19 (6.1)	11 (4.5)	14 (10.5)	6 (8.3)
Other Health Professions (*Diagnostic Imaging Technician, Speech Therapy, Dentistry, Health Psychology, Pharmacy, Podiatry, Occupational Therapy*)	56 (7.4)	20 (6.4)	26 (10.7)	7 (5.2)	3 (4.2)
Type of centre (*n* = 757)					
Hospital	395 (52)	134 (42.9)	146 (60.3)	67 (50.4)	48 (66.7)
Private Centre	129 (17)	79 (25.3)	28 (11.6)	19 (14.3)	3 (4.2)
Primary Care Centre	59 (7.8)	17 (5.4)	23 (9.5)	11 (8.3)	8 (11.1)
Residential Home	48 (6.3)	15 (4.8)	14 (5.8)	14 (10.5)	5 (6.9)
Emergency Department	44 (5.8)	24 (7.7)	5 (2.1)	8 (6.0)	7 (9.7)
Mutual Insurance Company	37 (4.9)	7 (2.2)	3 (1.2)	3 (2.3)	0 (0)
Clinic	13 (1.7)	19 (6.1)	11 (4.5)	6 (4.5)	1 (1.4)
Sports Club	11 (1.4)	8 (2.6)	2 (0.8)	1 (0.8)	0 (0)
Other types of centre (*Mental Health Centre, Educational Centre, Private Foundation, Epidemiological Surveillance, Sociosanitary Centre, Home‐based Therapy*)	21 (3.1)	9 (2.8)	9 (3.7)	4 (3.0)	0 (0)
Experience in years (*n* = 757)					
< 1 year	41 (5.4)	23 (7.4)	7 (2.9)	7 (5.3)	4 (5.6)
1–3 years	131 (17.3)	41 (13.1)	46 (19.0)	32 (24.1)	12 (16.7)
4–9 years	169 (22.3)	54 (61.9)	60 (24.8)	30 (22.6)	25 (34.7)
> 10 years	416 (55.0)	193 (61.9)	129 (53.3)	63 (47.4)	31 (43.1)

Abbreviation: BOS, Burnout Syndrome.

### 5.2 Testing Research Question

The prevalence of BOS represented a percentage of 58.9% (therefore concerning 447 participants), among which 242 participants (31.9%) showed mild level (one positive dimension among the three existing dimensions), 133 (17.5%) showed moderate level (two dimensions out of three) and 72 (9.5%) showed severe level of BOS (three positive dimensions). A total of 382 participants (50.3%) of the sample showed high EE, whereas 219 participants (28.9%) had high DP level, and 135 participants (17.8%) displayed low levels of PF.

Concerning the physical symptoms on HCPs, a total of 579 (76.3%) of the participants from the sample experienced muscle pain, whereas 525 (69.2%) showed unspecific pain in the vertebral region. Cephalea was present in 339 participants (44.7%) from the sample, whilst 33 (4.3%) participants reported vertigo and 7 (0.9%) participants had gastrointestinal symptoms.

Several potential relationships of the BOS with other sociodemographic and/or clinical variables were explored and revealed the existence of statistically significant differences: BOS and physical symptoms (χ^2^ = 40.46; *p* ≤ 0.001), BOS and muscle pain in the vertebral region (*χ* ^2^ = 46.77; *p* ≤ 0.001), and BOS and health professional area (*χ* ^2^ = 47.16; *p* ≤ 0.001).

With respect to the different subdimensions of the BOS, the Levene's test performed led to the application of the non‐parametric Kruskal–Wallis test to analyse the aforementioned subdimensions by gender (*p* = 0.017). Two subdimensions showed statistically significant differences per gender: women showed higher EE (*Z* = −3.46; *p* = 0.001), whilst men showed higher DP (*Z* = −2.69; *p* = 0.007). Non‐statistically significant differences were stated with respect to the subdimension PF per gender (*Z* = 0.041; *p* = 0.967). Concerning the aforementioned subdimensions, the Mann–Whitney *U*‐test performed between the means of prevalence by health profession (specifically between Nursing and Medicine, since both were the most frequent figures) revealed no statistically significant differences in regard to EE, DP and PF (*p* values of 0.618, 0.075 and 0.181, respectively).

**FIGURE 1:**
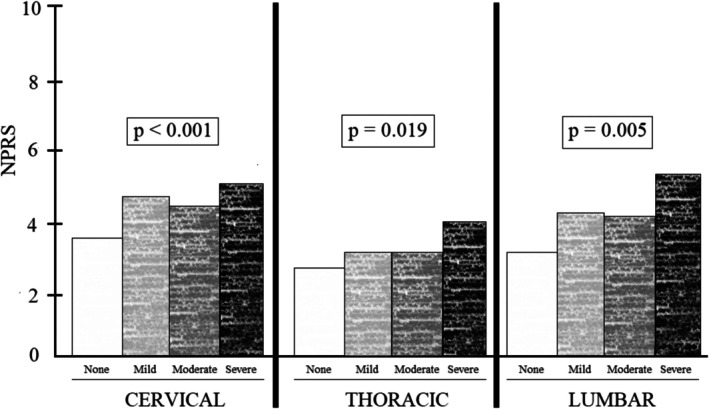
Numeric Pain Rating Scale (NPRS)‐assessed pain per vertebral region and level of Burnout Syndrome (BOS).

Table 2 displays the different levels of pain intensity according to the NPRS assessment of the different regions (cervical, thoracic and lumbar). An analysis of variance (ANOVA) test was used to compare the mean values of pain across the different groups by BOS‐stratum. The results reveal the presence of significant differences across the three NPRS assessments. The mean score of the CDI was 21.22 and standard deviation was 11.68 in a 0–100 range, which corresponds to a mild disability. Further visual information on the NPRS‐assessed level of pain per vertebral region is displayed in Figure 1.

**TABLE 2:** Levels of pain intensity (NPRS) and BOS levels (*n* = 759).

Vertebral regions	Presence of muscle pain (*n*, %)	NPRS pain	NPRS per BOS levels [range 0–10]				ANOVA across groups	Effect size *f*
		Mean and SD [range 0–10]	Absence	Mild	Moderate	Severe		
Cervical	525 (69.2%)	M = 4.36; SD = 2.07	M = 3.85; SD = 1.88	M = 4.68; SD = 2.10	M = 4.51; SD = 2.09	M = 4.77; SD = 2.21	*F* = 6.06; *p* ≤ 0.001	0.253
Thoracic	437 (57.6%)	M = 3.4; SD = 2.28	M = 2.95; SD = 2.19	M = 3.53; SD = 2.32	M = 3.53; SD = 2.22	M = 4.00; SD = 2.34	*F* = 3.34; *p* = 0.019	0.217
Lumbar	468 (61.7%)	M = 4.17; SD = 2.61	M = 3.63; SD = 2.47	M = 4.35; SD = 2.56	M = 4.24; SD = 2.55	M = 4.98; SD = 2.93	*F* = 4.35; *p* = 0.005	0.238

Abbreviations: BOS, Burnout Syndrome; NPRS, Numeric Pain Rating Scale.

The Pearson correlations performed revealed the existence of a linear relationship between the extent of cervical disability and the presence of BOS. The three subdimensions of BOS correlated with the corresponding CDI values, with the following results: moderate positive relationship between CDI and EE (*r* = 0.438; *p* ≤ 0.001), weak positive relationship between CDI and DP (*r* = 0.234; *p* ≤ 0.001) and weak negative relationship between CDI and PF (*r* = −0.256; *p* ≤ 0.001). The ANOVA test performed to compare the mean values of the CDI across the different BOS level led to the identification of statistically significant relationships across groups (*F* = 33.159; *p* ≤ 0.001).

The multinomial logistic regression performed with the absence of burnout as the reference category revealed age [*p* = 0.013; OR (95% CI) = 1.043 (1.009–1.079)], healthcare area of expertise (Nursing) [*p* = 0.031; OR (95% CI) = 1.135 (1.012–1.272)] and type of healthcare centre or setting (hospital or emergency department) [*p* = 0.001; OR (95% CI) = 1.231 (1.095–1.383)] as statistically significant variables concerning the development of mild level of burnout, whereas for the development of moderate and severe BOS solely the type of healthcare centre or setting emerged as a statistically significant variable (hospital or emergency Department) [*p* = 0.038; OR (95% CI) = 1.134 (1.007–1.277) and *p* = 0.010; OR (95% CI) = 1.177 (1.040–1.332), respectively].

## 6 Discussion

The results from the current study confirm similar prevalence rates of BOS among HCPs similar to those from other studies (Soto‐Rubio et al. 2020). Considering the severity or intensity of the BOS, a 31.9% of our sample showed mild BOS, a percentage of 17.5% corresponded to moderate BOS, and 9.5% of the sample had developed severe BOS. These results are in line with studies conducted in the same period (Soto‐Rubio et al. 2020) and show higher percentages than studies developed in previous contexts (Embriaco et al. 2012; Zarei et al. 2019; Lasalvia et al. 2021; Serdà et al. 2021). The main stressors linked to the delivery of healthcare reported correspond to the increase in the length of the workday, physical overload, the need to comply with strict regulations in terms of post‐COVID‐19 prevention measures, changes in the focus of the different healthcare professions with no previous training, and the fact of directly facing deceases and isolated patients with no family interaction (Gualano et al. 2021; Roslan et al. 2021; Serdà et al. 2021). This situation entails an increase in the degree of anxiety, depression and stress among HCPs, alongside the increase in the risk of suffering from BOS in the long term (Gündoğmuş et al. 2022; Lasalvia et al. 2021).

The results stemming from the current study suggest that sex is not a specific predictor of the development of BOS: this finding is nonetheless divergent and discrepant from other studies on this topic (Suñer‐Soler et al. 2013; Van Hoy et al. 2022). On another note, considering the different dimensions of the BOS questionnaire and analysing per sex, the current study determines that women health professionals with BOS show higher EE, whereas men professionals tend to develop higher DP (Chen et al. 2021). This result could confirm than men and women experience BOS in different ways, and the psychological therapeutic approach on this condition could diverge depending on the gender and the affected dimension of BOS. Higher EE among women could be due to higher comparative prevalence rates of depression and anxiety in women versus men; the pandemic situation has created a work environment fostering the exacerbation of symptoms of EE (Lasalvia et al. 2021). On another note, the DP is usually channelled through cynicism and mental estrangement towards patients, a fact that generates maladaptive mechanisms of cognitive emotional regulation to the stressful situation which, in turn, may increase the risk of DP when sustained over time (López‐López et al. 2019).

This study displays similar rates of BOS across nurses, nursing assistants and medical doctors. This result was somehow unexpected and counterintuitive, since it diverges from previous studies showing lower percentages of BOS among medical doctors compared to nurses (Chen‐Lim et al. 2022; Shultz et al. 2016). Despite the similarity in the prevalence of BOS across the three professional categories (nurses, medical doctors, nursing assistants), the logistic regression analysis performed identified the profile of nurses as the professional figure at higher risk of suffering from BOS. Focusing on a specific profile specifically sensitive to the development of BOS, age was directly related to the BOS, to wit, the younger the health professional, the higher the probability of developing mild BOS is (Tsai and Tsou 2022; Youssef et al. 2022). Despite the fact that the odds‐ratio is relatively low, for every 1‐year increase in terms of age, the odds of developing mild BOS decreases by 4%.

As for the different dimensions of BOS based on the professional category, this study determines that nurses usually show high EE and low PF. On another note, medical doctors tend to develop higher DP. No similar studies have been identified in this respect. A feasible explanation to these results could be based on the fact that nurses independently of sex variable usually have a sustained and first‐line contact with patients, with long workdays and they are subsequently more prone to the fact of facing more traumatic experiences leading to the development of feelings of exhaustion generating EE (López‐López et al. 2019; Mo et al. 2020) whilst professionals in the field of Medicine have to make extreme and vital decisions with a high psycho‐emotional impact entailing high odds of increasing their degree of DP.

The results from the current study show that the presence of BOS is associated with physical symptoms, such as muscle pain (Tsai and Tsou 2022). In this conceptual framework, and delving into the binomial BOS–muscle pain, a positive psycho‐somatic relationship was stated in the study presented. The analysis performed confirms a higher number of physical symptoms in professionals with BOS, with respect to professionals not having developed BOS. Other studies have confirmed the relationship between unspecific muscle pain and psychological symptoms as distress and anxiety (Balagué et al. 2012; Cunill et al. 2020; Ortego et al. 2016). Professionals showing mild or moderate BOS tended to identify muscle pain in the cervical region whereas, for professionals showing severe BOS, the vertebral region with higher pain intensity corresponded to the lumbar or low‐back region. The more severe the BOS, the higher the cervical disability is. In the same line, the higher the EE and DP, and the lower the PF, the more severe the cervical disability is.

### 6.1 Limitations

Our findings should be interpreted in the light of the study limitations: first, the sample corresponded to a convenience sampling, a fact that limits the knowledge of real sample distribution. Furthermore, our study corresponds to a cross‐sectional design, which implies that our findings could lack of stability across time. Secondly, the regression analysis performed revealed a Nagelkerke's *R*‐squared of the model of 0.113, which implies that the independent variables did not explain much in the variation of the dependent variable of the model (i.e., the predictors solely explain around 11% of the variation of the dependent response variable): the possible inclusion of further variables could improve the *R*‐squared value, but a subsequent analysis for multicollinearity in the variables analysed revealed acceptable values, with a final tolerance of 0.3 and a variance inflation factor 3.33. Third, data on possible comorbidities that may contribute to the occurrence of pain in the cervical spine have not been taken into account: further studies shall consider the co‐presence of comorbidities interfering with or having a potential impact on pain in the cervical region.

### 6.2 Recommendations for Further Research

Based on the results of this study, younger HCPs are at a higher risk of BOS than older HCPs, while sex did not have an impact on this matter (West et al. 2018). Furthermore, nurses are the most sensitive and vulnerable group, whilst working in a hospital and/or emergency department are also especially sensitive areas (López‐López et al. 2019). Thus, the professional profile of a nurse of young age working in a hospital and/or emergency department emerges as a specific vulnerable profile and clearly highlights the importance of prioritising tailored preventative or therapeutic interventions to avoid and/or decrease the development of BOS on these specific profiles. As for the development of higher level of BOS, the results disclose the paramount importance of the setting (hospital or emergency department) and therefore point out the importance of the professional centre as a key element for developing moderate and severe syndromes. Further research should focus on the development and implementation of multidimensional tailored interventions to decrease and/or revert symptoms from BOS, including psychological aspects associated to the syndrome itself, alongside decreasing unspecific muscle pain in the vertebral region (Alenezi et al. 2019). Further programs focusing on specific on preventive strategies shall be implemented and channelled through (1) global organisational strategies (institutionally addressing psychosocial risk factors, providing reasonable work accommodations for workers with mental health conditions, as well as prioritising family‐friendly working time arrangements, enhancing health facility performance, or facilitating worker choice and influence over their hours of work, among others), and (2) personal tailored interventions (using universally delivered psychosocial interventions, encouraging physical activity), as already endorsed and extolled by international organisations as the World Health Organization (WHO 2018) or the International Labor Organization (ILO 2018).

## 7 Conclusion

The high prevalence of BOS detected among HCPs propels stakeholders to take proactive actions to prevent and revert this health condition, especially considering the profile of nurse HCPs of young age working at a hospital or emergency department, since this specific profile is at higher risk of suffering from BOS. The current study therefore confirms the need to prevent and overcome BOS in HCPs.

## Author Contributions

Study conception and design: Núria Puigtió‐Rebollo, Bernat Carles Serdà‐Ferrer and Miquel Sitjar Suñer. Data collection: Núria Puigtió‐Rebollo and Bernat Carles Serdà‐Ferrer. Data analysis and interpretation: Núria Puigtió‐Rebollo, Bernat Carles Serdà‐Ferrer and Mariano Gacto‐Sánchez. Drafting of the article: Núria Puigtió‐Rebollo, Bernat Carles Serdà‐Ferrer, Mariano Gacto‐Sánchez and Miquel Sitjar Suñer. Critical revision of the article: Núria Puigtió‐Rebollo, Bernat Carles Serdà‐Ferrer and Mariano Gacto‐Sánchez.

## Disclosure

Mariano Gacto‐Sánchez is a lecturer and researcher in Biostatistics applied to Physiotherapy.

## Conflicts of Interest

The authors declare no conflicts of interest.

## Data Availability

The data that support the findings of this study are available from the corresponding author upon reasonable request.

## References

[nop270379-bib-0001] Alenezi, A. , S. McAndrew , and P. Fallon . 2019. “Burning out Physical and Emotional Fatigue: Evaluating the Effects of a Programme Aimed at Reducing Burnout Among Mental Health Nurses.” International Journal of Mental Health Nursing 28, no. 5: 1045–1055. 10.1111/INM.12608. 31231965

[nop270379-bib-0002] Ali, S. A. O. , and A. K. A. Eissa . 2018. “Relation Between Burnout and Psychosomatic Symptoms Among Staff Nursing in Intensive Care Units.” Malaysian Journal of Nursing (MJN) 9, no. 4: 20–28.

[nop270379-bib-0003] Balagué, F. , A. F. Mannion , F. Pellisé , and C. Cedraschi . 2012. “Non‐Specific Low Back Pain.” Lancet 379, no. 9814: 482–491. 10.1016/S0140-6736(11)60610-7. 21982256

[nop270379-bib-0004] Bamforth, K. , P. Rae , J. Maben , H. Lloyd , and S. Pearce . 2023. “Perceptions of Healthcare Professionals' Psychological Wellbeing at Work and the Link to Patients' Experiences of Care: A Scoping Review.” International Journal of Nursing Studies Advances 1, no. 5: 100148. 10.1016/j.ijnsa.2023.100148. PMC11080414 38746580

[nop270379-bib-0005] Baro Vila, R. C. , L. M. Burgos , A. Sigal , J. P. Costabel , and A. Alves de Lima . 2022. “Burnout Syndrome in Cardiology Residents. Impact of the COVID‐19 Pandemic on Burnout Syndrome in Cardiology Residents.” Current Problems in Cardiology 47, no. 1: 100873. 10.1016/J.CPCARDIOL.2021.100873. 34108084 PMC8612459

[nop270379-bib-0006] Chen, R. , C. Sun , J.‐J. Chen , et al. 2021. “A Large‐Scale Survey on Trauma, Burnout, and Posttraumatic Growth Among Nurses During the COVID‐19 Pandemic.” International Journal of Mental Health Nursing 30, no. 1: 102–116. 10.1111/INM.12796. 33107677 PMC7894338

[nop270379-bib-0007] Chen‐Lim, M. L. , M. A. McCabe , H. Xu , et al. 2022. “Experiences of U.S. Nurses Compared With Nonnurses in the First Year of COVID‐19: Findings From a National Registry.” Nursing Research 71, no. 6: 421–431. 10.1097/NNR.0000000000000610. 35878076 PMC9640262

[nop270379-bib-0008] Cunill, M. , M. Aymerich , B. C. Serdà , and J. Patiño‐Masó . 2020. “The Impact of COVID‐19 on Spanish Health Professionals: A Description of Physical and Psychological Effects.” International Journal of Mental Health Promotion 22, no. 3: 185–198. 10.32604/IJMHP.2020.011615.

[nop270379-bib-0009] De Hert, S. 2020. “Burnout in Healthcare Workers: Prevalence, Impact and Preventative Strategies.” Local and Regional Anesthesia 13: 171–183. 10.2147/LRA.S240564. 33149664 PMC7604257

[nop270379-bib-0010] Dyrbye, L. N. , C. P. West , D. Satele , et al. 2014. “Burnout Among u.s. Medical Students, Residents, and Early Career Physicians Relative to the General u.s. Population.” Academic Medicine 89, no. 3: 443–451. 10.1097/ACM.0000000000000134. 24448053

[nop270379-bib-0011] Embriaco, N. , E. Azoulay , K. Barrau , et al. 2012. “High Level of Burnout in Intensivists.” 175, no. 7: 686–692. 10.1164/Rccm.200608-1184OC. 17234905

[nop270379-bib-0012] Gil‐Monte, P. R. 2005. “Factorial Validity of the Maslach Burnout Inventory (MBI‐HSS) Among Spanish Professionals.” Revista de Saúde Pública 39, no. 1: 1–8. 10.1590/S0034-89102005000100001. 15654454

[nop270379-bib-0013] Giordano, F. , A. Cipolla , and M. Ungar . 2022. “Building Resilience for Healthcare Professionals Working in an Italian Red Zone During the COVID‐19 Outbreak: A Pilot Study.” Stress and Health 38, no. 2: 234–248. 10.1002/SMI.3085. 34312986 PMC9292917

[nop270379-bib-0014] Gualano, M. R. , T. Sinigaglia , G. Lo Moro , et al. 2021. “The Burden of Burnout Among Healthcare Professionals of Intensive Care Units and Emergency Departments During the COVID‐19 Pandemic: A Systematic Review.” International Journal of Environmental Research and Public Health 18, no. 15: 8172. 10.3390/IJERPH18158172. 34360465 PMC8346023

[nop270379-bib-0015] Gündoğmuş, İ. , C. Ünsal , M. Filiz , et al. 2022. “Post‐Traumatic Stress Disorder Symptoms, Psychosomatic Symptoms, and Sleep Quality in Health Care Workers in Turkey During the COVID‐19 Outbreak: Data From a Large Tertiary Care Hospital.” Psychiatric Annals 52, no. 10: 427–441. 10.3928/00485713-20220928-01.

[nop270379-bib-0016] Herr, K. A. , K. Spratt , P. R. Mobily , and G. Richardson . 2004. “Pain Intensity Assessment in Older Adults: Use of Experimental Pain to Compare Psychometric Properties and Usability of Selected Pain Scales With Younger Adults.” Clinical Journal of Pain 20, no. 4: 207–219. 10.1097/00002508-200407000-00002. 15218405

[nop270379-bib-0017] International Labor Organization . 2018. “Decent Working Time for Nursing Personnel: Critical for Worker Well‐Being and Quality Care.” https://www.ilo.org/publications/decent‐working‐time‐nursing‐personnel‐critical‐worker‐well‐being‐and.

[nop270379-bib-0018] Ivánkovits, L. , C. Kazinczi , K. Kocsis , et al. 2024. “A Simplified Measure of Burnout Symptoms Among Paramedics—An Exploratory Analysis of a Hungarian Sample.” BMC Psychology 12: 37. 10.1186/s40359-024-01518-x. 38238830 PMC10797803

[nop270379-bib-0019] Jensen, M. P. , and C. A. McFarland . 1993. “Increasing the Reliability and Validity of Pain Intensity Measurement in Chronic Pain Patients.” Pain 55, no. 2: 195–203. 10.1016/0304-3959(93)90148-I. 8309709

[nop270379-bib-0020] Lasalvia, A. , L. Bodini , F. Amaddeo , et al. 2021. “The Sustained Psychological Impact of the COVID‐19 Pandemic on Health Care Workers One Year After the Outbreak—A Repeated Cross‐Sectional Survey in a Tertiary Hospital of North‐East Italy.” International Journal of Environmental Research and Public Health 18, no. 24: 13374. 10.3390/IJERPH182413374. 34948981 PMC8707618

[nop270379-bib-0021] Liu, X. , J. Chen , D. Wang , et al. 2020. “COVID‐19 Outbreak Can Change the Job Burnout in Health Care Professionals.” Frontiers in Psychiatry 11: 1362. 10.3389/FPSYT.2020.563781/BIBTEX. PMC7753007 33363480

[nop270379-bib-0022] López‐López, I. M. , J. L. Gómez‐Urquiza , G. R. Cañadas , E. I. De la Fuente , L. Albendín‐García , and G. A. Cañadas‐De la Fuente . 2019. “Prevalence of Burnout in Mental Health Nurses and Related Factors: A Systematic Review and Meta‐Analysis.” International Journal of Mental Health Nursing 28, no. 5: 1035–1044. 10.1111/INM.12606. 31132216

[nop270379-bib-0023] MacDermid, J. C. , D. M. Walton , S. Avery , et al. 2009. “Measurement Properties of the Neck Disability Index: A Systematic Review.” Journal of Orthopaedic & Sports Physical Therapy 39, no. 5: 400–416. 10.2519/JOSPT.2009.2930. 19521015

[nop270379-bib-0046] Maslach, C. , and S. E. Jackson . 1986. Maslach Burnout Inventory Manual, 2nd ed. Consulting Psychologists Press.

[nop270379-bib-0024] Mo, Y. , L. Deng , L. Zhang , et al. 2020. “Work Stress Among Chinese Nurses to Support Wuhan in Fighting Against COVID‐19 Epidemic.” Journal of Nursing Management 28, no. 5: 1002–1009. 10.1111/JONM.13014. 32255222 PMC7262235

[nop270379-bib-0025] Ortego, G. , J. H. Villafañe , V. Doménech‐García , P. Berjano , L. Bertozzi , and P. Herrero . 2016. “Is There a Relationship Between Psychological Stress or Anxiety and Chronic Nonspecific Neck‐Arm Pain in Adults? A Systematic Review and Meta‐Analysis.” Journal of Psychosomatic Research 90: 70–81. 10.1016/J.JPSYCHORES.2016.09.006. 27772562

[nop270379-bib-0027] Rizzo, A. , M. Yıldırım , G. G. Öztekin , et al. 2023. “Nurse Burnout Before and During the COVID‐19 Pandemic: A Systematic Comparative Review.” Frontiers in Public Health 11: 1225431. 10.3389/fpubh.2023.1225431. 37732086 PMC10507882

[nop270379-bib-0028] Roslan, N. S. , M. S. B. Yusoff , A. R. Asrenee , and K. Morgan . 2021. “Burnout Prevalence and Its Associated Factors Among Malaysian Healthcare Workers During COVID‐19 Pandemic: An Embedded Mixed‐Method Study.” Healthcare (Basel) 9, no. 1: 90. 10.3390/HEALTHCARE9010090. 33477380 PMC7829836

[nop270379-bib-0045] Schaufeli, W. B. , and E. R. Greenglass . 2001. “Introduction to Special Issue on Burnout and Health.” Psychology & Health 16, no. 5: 501–510. 10.1080/08870440108405523. 22804495

[nop270379-bib-0029] Serdà, B. C. , M. Aymerich , J. Patiño‐Masó , and M. Cunill . 2021. “Mental Health Screening of Healthcare Professionals Who Are Candidates for Psychological Assistance During the COVID‐19 Pandemic.” International Journal of Environmental Research and Public Health 18, no. 21: 11167. 10.3390/IJERPH182111167. 34769686 PMC8583048

[nop270379-bib-0030] Shultz, J. M. , J. L. Cooper , F. Baingana , et al. 2016. “The Role of Fear‐Related Behaviors in the 2013–2016 West Africa Ebola Virus Disease Outbreak.” Current Psychiatry Reports 18, no. 11: 1–14. 10.1007/S11920-016-0741-Y/METRICS. 27739026 PMC5241909

[nop270379-bib-0031] Soto‐Rubio, A. , M. D. C. Giménez‐Espert , and V. Prado‐Gascó . 2020. “Effect of Emotional Intelligence and Psychosocial Risks on Burnout, Job Satisfaction, and Nurses' Health During the COVID‐19 Pandemic.” International Journal of Environmental Research and Public Health 17, no. 21: 7998. 10.3390/IJERPH17217998. 33143172 PMC7663663

[nop270379-bib-0032] Suñer‐Soler, R. , A. Grau‐Martín , S. Font‐Mayolas , M. E. Gras , C. Bertran , and M. J. M. Sullman . 2013. “Burnout and Quality of Life Among Spanish Healthcare Personnel.” Journal of Psychiatric and Mental Health Nursing 20, no. 4: 305–313. 10.1111/J.1365-2850.2012.01897.X. 22404294

[nop270379-bib-0033] Surendran, A. , L. Beccaria , S. Rees , and P. Mcilveen . 2024. “Cognitive Mental Workload of Emergency Nursing: A Scoping Review.” Nursing Open 11, no. 2: e2111. 10.1002/nop2.2111. 38366782 PMC10873679

[nop270379-bib-0034] Tsai, H. J. , and M. T. Tsou . 2022. “Age, Sex, and Profession Difference Among Health Care Workers With Burnout and Metabolic Syndrome in Taiwan Tertiary Hospital—A Cross‐Section Study.” Frontiers in Medicine 9: 854403. 10.3389/FMED.2022.854403. 35492349 PMC9048413

[nop270379-bib-0035] Van Hoy, A. , M. Rzeszutek , M. Pięta , et al. 2022. “Burnout Among Psychotherapists: A Cross‐Cultural Value Survey Among 12 European Countries During the Coronavirus Disease Pandemic.” Scientific Reports 12, no. 1: 1–11. 10.1038/s41598-022-17669-z. 35941352 PMC9358385

[nop270379-bib-0036] Vernon, H. 2008. “The Neck Disability Index: State‐of‐the‐Art, 1991–2008.” Journal of Manipulative and Physiological Therapeutics 31, no. 7: 491–502. 10.1016/j.jmpt.2008.08.006. 18803999

[nop270379-bib-0037] Wang, J. , W. Wang , S. Laureys , and H. Di . 2020. “Burnout Syndrome in Healthcare Professionals Who Care for Patients With Prolonged Disorders of Consciousness: A Cross‐Sectional Survey.” BMC Health Services Research 20, no. 1: 841. 10.1186/s12913-020-05694-5. 32894132 PMC7487695

[nop270379-bib-0038] West, C. P. , L. N. Dyrbye , and T. D. Shanafelt . 2018. “Physician Burnout: Contributors, Consequences and Solutions.” Journal of Internal Medicine 283, no. 6: 516–529. 10.1111/JOIM.12752. 29505159

[nop270379-bib-0039] World Health Organization . 2018. “Psychosocial Risks and Mental Health.” https://www.who.int/tools/occupational‐hazards‐in‐health‐sector/psycho‐social‐risks‐mental‐health.

[nop270379-bib-0040] Wu, P. L. , S. M. Tseng , Y. C. Tseng , L. C. Chen , H. C. Pai , and W. J. Yen . 2021. “Job Stress and Occupational Burnout Among Clinical Nursing Teachers: A Cross‐Sectional Study.” Journal of Professional Nursing 37, no. 5: 907–915. 10.1016/J.PROFNURS.2021.07.014. 34742521

[nop270379-bib-0041] Youssef, D. , J. Youssef , L. Abou‐Abbas , M. Kawtharani , and H. Hassan . 2022. “Prevalence and Correlates of Burnout Among Physicians in a Developing Country Facing Multi‐Layered Crises: A Cross‐Sectional Study.” Scientific Reports 12, no. 1: 12615. 10.1038/S41598-022-16095-5. 35871153 PMC9308770

[nop270379-bib-0042] Zarei, E. , F. Ahmadi , M. S. Sial , J. Hwang , P. A. Thu , and S. M. Usman . 2019. “Prevalence of Burnout Among Primary Health Care Staff and Its Predictors: A Study in Iran.” International Journal of Environmental Research and Public Health 16, no. 12: 2249. 10.3390/IJERPH16122249. 31242691 PMC6616853

[nop270379-bib-0043] Zhang, W. , R. Miao , J. Tang , et al. 2021. “Burnout in Nurses Working in China: A National Questionnaire Survey.” International Journal of Nursing Practice 27, no. 6: e12908. 10.1111/ijn.12908. 33336456

[nop270379-bib-0044] Zhou, T. , C. Xu , C. Wang , et al. 2022. “Burnout and Well‐Being of Healthcare Workers in the Post‐Pandemic Period of COVID‐19: A Perspective From the Job Demands‐Resources Model.” BMC Health Services Research 22, no. 1: 1–15. 10.1186/S12913-022-07608-Z/FIGURES/4. 35236354 PMC8888816

